# Consensus Molecular Subtypes Efficiently Classify Gastric Adenocarcinomas and Predict the Response to Anti-PD-1 Immunotherapy

**DOI:** 10.3390/cancers14153740

**Published:** 2022-07-31

**Authors:** Xiangyan Wu, Yuhan Ye, Kenneth J. Vega, Jiannan Yao

**Affiliations:** 1 Key Laboratory of Gastrointestinal Cancer (Fujian Medical University), Ministry of Education, Fuzhou 350122, China; xiangyanwu@fjmu.edu.cn; 2 Fujian Key Laboratory of Tumor Microbiology, Department of Medical Microbiology, Fujian Medical University, Fuzhou 350122, China; 3 Department of Pathology, Zhongshan Hospital, Xiamen University, Xiamen 361004, China; 23120150150699@stu.xmu.edu.cn; 4 Department of Gastroenterology and Hepatology, Augusta University, Augusta, GA 30912, USA; kvega@augusta.edu; 5 Department of Oncology, Beijing Chao-Yang Hospital, Capital Medical University, Beijing 100020, China

**Keywords:** molecular characteristic, tumor microenvironment, pembrolizumab, microsatellite instability, epithelial–mesenchymal transition, companion biomarker

## Abstract

**Simple Summary:**

Gastric adenocarcinoma (GAC) is most commonly classified based on a system developed by the Cancer Genome Atlas in 2014. However, this subtyping system cannot efficiently identify suitable candidates for immunotherapy. Because GAC is highly heterogeneous and closely related to CRC at the molecular and functional levels, we explored the clinical utility of CMS classification originally developed for CRC and found that the CMS subtyping system can efficiently classify GAC. CMS1-4 classifications in GAC recapitulated their corresponding CRC subtype characteristics. Notably, CMS1 predicted a favorable response to anti-PD-1 therapy, and CMS4 outperformed the classical TCGA subtyping prognostic prediction and identified patients with an unfavorable anti-PD-1 response. Strikingly, partitioning the CMS4 subtype by EMT activation identified an additional anti-PD-1-susceptible patient subgroup. These results provide new insights that may help to improve clinical outcomes in immunotherapy candidates.

**Abstract:**

Background: Gastric adenocarcinoma (GAC) is highly heterogeneous and closely related to colorectal cancer (CRC) both molecularly and functionally. GAC is currently subtyped using a system developed by TCGA. However, with the emergence of immunotherapies, this system has failed to identify suitable treatment candidates. Methods: Consensus molecular subtypes (CMSs) developed for CRC were used for molecular subtyping in GAC based on public expression cohorts, including TCGA, ACRG, and a cohort of GAC patients treated with the programmed cell death 1 (PD-1) inhibitor pembrolizumab. All aspects of each subtype, including clinical outcome, molecular characteristics, oncogenic pathway activity, and the response to immunotherapy, were fully explored. Results: CMS classification was efficiently applied to GAC. CMS4, characterized by EMT activation, stromal invasion, angiogenesis, and the worst clinical outcomes (median OS 24.2 months), was the predominant subtype (38.8%~44.3%) and an independent prognostic indicator that outperformed classical TCGA subtyping. CMS1 (20.9%~21.5%) displayed hypermutation, low SCNV, immune activation, and best clinical outcomes (median OS > 120 months). CMS3 (17.95%~25.7%) was characterized by overactive metabolism, KRAS mutation, and intermediate outcomes (median OS 85.6 months). CMS2 (14.6%~16.3%) was enriched for WNT and MYC activation, differentiated epithelial characteristics, APC mutation, lack of ARID1A, and intermediate outcomes (median OS 48.7 months). Notably, CMS1 was strongly correlated with immunotherapy biomarkers and favorable for the anti-PD-1 drug pembrolizumab, whereas CMS4 was poorly responsive but became more sensitive after EMT-based stratification. Conclusions: Our study reveals the practical utility of CMS classification for GAC to improve clinical outcomes and identify candidates who will respond to immunotherapy.

## 1. Introduction

Molecular subtyping is emerging as a means to identify at-risk patients and personalize therapy in a variety of tumor types [1]. Most notably, molecular subtyping schemes have become a mainstay of breast cancer treatment, enabling the avoidance of ineffective treatment regimens and limiting the use of chemotherapy and/or maintenance therapies with significant and unpleasant side effects [2,3,4]. Recently, a variety of marker types have been demonstrated that identify tumors susceptible to anti-PD1- and anti-PD-L1-based immunotherapies and, consequently, improve patient outcomes [5,6].

Although several molecular alterations, such as microsatellite instability (MSI) [7] and chromosomal instability (CIN) [8], provide insight into colorectal cancer (CRC) tumorigenesis, their clinical utility is limited by discrepant results. To overcome these issues, a unified and standardized transcriptomic classification of CRC into four consensus molecular subtypes (CMSs) was developed [9]. CMS1 contains mostly microsatellite instable (MSI) tumors with hypermutation/hypermethylation and strong immune activation. CMS2 presents with CIN, as well as upregulation of WNT and MYC signaling. CMS3 is enriched for KRAS mutations and metabolically overactivated. CMS4 is defined by TGF-beta pathway activation, epithelial–mesenchymal transition (EMT), and angiogenesis [9]. Since publication, CMS has been used in multiple clinical trials and retrospective analyses of CRC, including CALGB/SWOG 80405 [10] and FOLFIRI, plus cetuximab or bevacizumab in the FIRE3 (AIO KRK-0306) trial [11], with results highlighting the potential clinical utility of CMS.

Currently, gastric adenocarcinoma (GAC) is most commonly classified based on a system developed by the Cancer Genome Atlas (TCGA) in 2014 [12,13]. This system divides tumors into four types, including the diffuse, genomically stable subtype (GS), the p53 mutant chromosomal instable subtype (CIN), the hypermutant microsatellite instability high subtype (MSI-H), and the Epstein–Barr virus-related subtype characterized by PI3K mutation and PD-L1/2 amplification (EBV). GAC is closely related to CRC at the molecular and functional levels [14]. Shared traits between GAC and CRC include specialized cell types in the tissue of origin (LGR5 stem cells, goblet cells, and sensory tuft cells) [15,16], loss of the APC tumor suppressor [17], activation of WNT signaling [18], and TGF-beta/EMT activation, among others [15,16,19,20,21]. Notably, assessment of the microenvironmental context across GI cancer types reveals a site-specific context for immunotherapy susceptibility characteristics, including microsatellite instability (MSI) [22]. Therefore, the application of CRC-derived markers to GAC is potentially feasible and worthy of serious investigation.

The treatment of metastatic GAC has evolved through several decades, primarily involving tolerance of chemotherapeutic toxicity in exchange for improved responses until the ToGA trial, which identified HER2 as a pioneering molecular target [23]. Subsequently, other targeted strategies for VEGFR, EGFR, FGFR, KIT, c-Met, and additional novel HER2-targeted agents began to emerge [24,25]. In the past decade, immunotherapy has revolutionized the clinical treatment of several solid tumors by providing durable responses and drastic increases in overall survival among select subsets of patients [26,27]. However, due to intratumoral and intertumoral heterogeneity, monotherapies against specific targets, including immunotherapies, often demonstrate limited responses [28]. Systemic therapies, including the combination of immunotherapy with standard chemotherapy or targeted therapy, are emerging as encouraging antitumor strategies. Additionally, notable agents targeting essential cellular components, such as tumor-associated macrophages, lymphocytes, cancer-associated fibroblasts, and mesenchymal stem cells, are currently under investigation to prime the tumor microenvironment (TME) for therapy, including immunotherapies. Importantly, there may be a synergistic benefit to developing multitarget immunotherapy regimens [29].

Among several immunotherapies, the anti-PD1 monoclonal antibody pembrolizumab is the first to be approved as a subsequent line of therapy for unresectable advanced, recurrent, or metastatic disease characterized by MSI-H/dMMR, PD-L1 combined positive score (CPS) ≥ 1, or tumor mutational burden (TMB) ≥ 10 mutations/megabases [30]. Pembrolizumab was also recently approved in combination with trastuzumab and chemotherapy as a first-line treatment for patients with locally advanced unresectable or metastatic HER2-positive GAC [31,32,33]. Among the TCGA classifications, there is evidence to suggest that MSI-H and EBV subtypes are more likely to be susceptible to approved immune checkpoint inhibitors (ICIs), such as pembrolizumab [34]. However, EBV is rarely tested in clinical practice, and even among immunologically “hot” tumors, the efficacy of ICI therapy ranges from 20 to 30% [35,36]. As a result, there is a pressing need for advanced companion biomarkers that can more effectively predict GAC patient responses to ICIs. Herein, we describe the application of the CMS classification to GAC and show that it can be used to identify the patients most likely to respond to pembrolizumab therapy.

## 2. Methods

### 2.1. Datasets and Processing of RNA Sequencing Reads

Paired-end FASTQ data for GAC patients treated with the programmed cell death 1 (PD-1) inhibitor pembrolizumab (*n* = 45) were obtained from the European Nucleotide Archive (ENA: PRJEB25780), and reads were trimmed using Trimmomatic to cut adapter and other Illumina-specific sequences from the reads. Trimmed reads were aligned to the human genome (hg38) using HiSat2, and counts were obtained using FeatureCounts. Copy number profile, RNA-seq, DNA methylation, and miRNA-seq data from TCGA gastric adenocarcinoma (STAD) and colorectal cancer (CRC) datasets were obtained from the University of California, Santa Cruz (UCSC), Cancer Genome Browser (https://xenabrowser.net) [37]. The expression profiling of the Asian Cancer Research Group (ACRG) study of gastric tumors (GSE62254; *n* = 300) was downloaded from the NCBI Gene Expression Omnibus (GEO). Poorly expressed genes were removed from transcriptome data when the average count per million (cpm) value was <1 or expression was not present in multiple tumors. Demographic and clinical characteristics for STAD (*n* = 415; Table S1) and CRC (*n* = 380) were obtained from the BROAD Institute’s GDAC (http://gdac.broadinstitute.org) [38]. For ACRG patients, demographic and clinical characteristics were obtained from GEO and related studies [39]. For pembrolizumab-treated GAC patients, demographic and clinical information was obtained from the associated study [34]. The basic information of all the datasets mentioned above is listed in Table 1.

### 2.2. Consensus Molecular Subtypes and Principal Component Analysis

CMS classifications were performed using the CMSCaller R package [40]. Principal component analyses (PCAs) were performed using the prcomp R function. STAD and CRC RNA-seq data were batch-corrected using the SVA R package, combined, and subjected to PCA.

### 2.3. Estimation of Tumor Cellular Components and EMT Activation

Immune score, stromal score, and tumor purity were calculated using the ESTIMATE R package [41] (Table S2). Tissue-infiltrating immune and stromal cell portions were quantified in TIMER 2.0 (http://timer.cistrome.org) based on xCell [42] and MCP-counter [43]. EMT activation was calculated by subtracting the mean value of mRNA expression for EMT-negative genes from the mean value of EMT-positive genes (Table S3).

### 2.4. Clinical Markers of Immunotherapy Response

The T-cell gene expression profile (GEP) was calculated by the mRNA expression of 18 genes introduced by Ayers et al. [44], including TIGIT, CD27, CD8A, PDCD1LG2 (PD-L2), LAG3, CD274 (PD-L1), CXCR6, CMKLR1, NKG7, CCL5, PSMB10, IDO1, CXCL9, HLA-DQA1, CD276, STAT1, HLA-DRB1, and HLA-E. Immune checkpoints were calculated by the mRNA expression of 7 genes listed by Mariathasan et al. [45], which included CD274, PDCD1LG2, CTLA4, PDCD1, LAG3, HAVCR2, and TIGIT.

### 2.5. Pathway Analysis

Pathway analyses were performed using PARADIGM algorithm-integrated pathways obtained from the UCSC Cancer Genome Browser and Gene Set Enrichment Analysis (GSEA) software v3. Enrichment scores are provided in Table S4.

### 2.6. Statistical Analysis

All statistical analyses were performed in R v 3.6.3. Comparison of each CMS classification with others was conducted by the Wilcoxon test for two-group comparison and Kruskal–Wallis test for comparisons of more than two groups; a *p* value < 0.05 was considered statistically significant. Kaplan–Meier and Cox regression analyses were performed for overall survival of STAD and ACRG, as well as subtypes of gastric cancer in pembrolizumab-treated patients, and visualized using the survival and survminer packages in R. Fisher’s exact test was used for contingency analysis of clinical and demographic factors. ROC analysis was performed to estimate the diagnostic ability of CMS classifications as an indication of overall survival and to estimate the ability of the CMS1 classification as an indication of PD-1 inhibitor response.

## 3. Results

### 3.1. Gastric Adenocarcinomas Are Discretely Classified by CRC Consensus Molecular Subtypes

**Figure 1:**
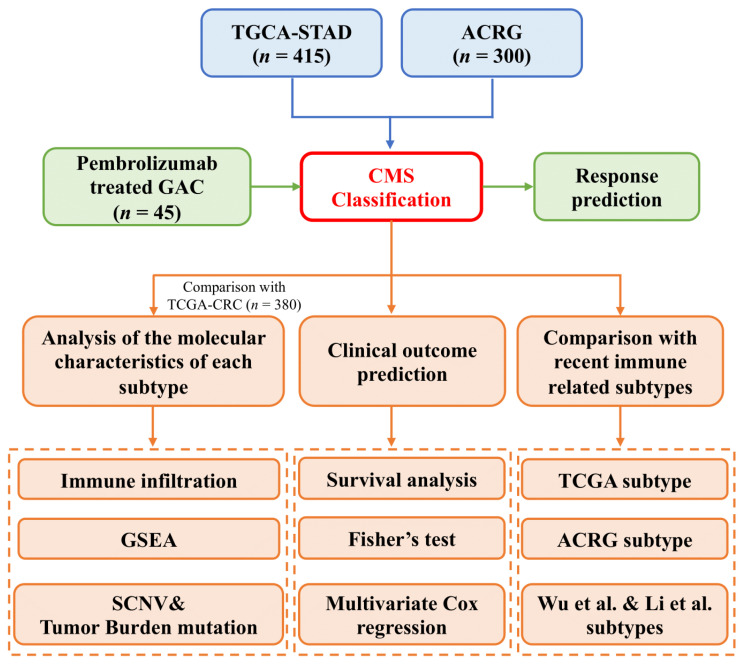
Analytical workflow showing the overall design of the study.

**Figure 2:**
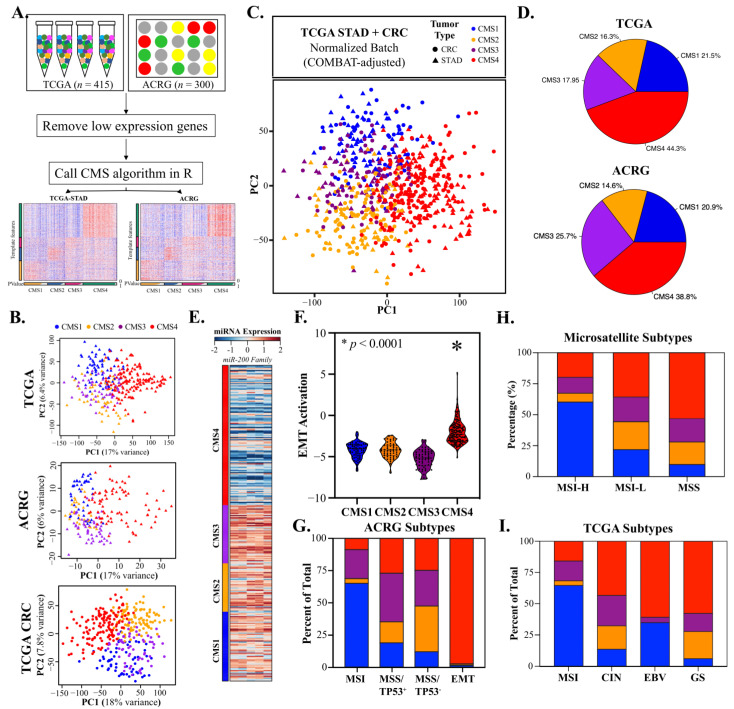
Consensus molecular subtypes (CMSs) can effectively classify gastric adenocarcinomas (GACs). (**A**). GAC RNA-Seq count data were downloaded from the TCGA (STAD; n = 415) and ACRG (n = 300) projects, filtered to exclude low-expression genes, and used to call CMS subtypes. (**B**). Principal component analysis (PCA) demonstrated that GAC could be divided into discrete groups based on CMS subtypes comparable to CRC (n = 380). (**C**). Application of batch correction to the TCGA GAC and CRC datasets using SVA followed by PCA showed homogeneity between GAC and CRC CMS classifications. (**D**). CMS4 is the most common classifier in GAC, followed by CMS1, CMS3, and CMS2. (**E**,**F**). CMS4 is strongly associated with downregulation of the miR-200 EMT inhibitor family and consequent activation of EMT. (**G**–**I**). CMS classifications demonstrate meaningful overlap but are distinct from current GAC classifications. Notably, CMS4 has strong contingency with the ACRG EMT subtype, and CMS1 has strong contingency for the microsatellite instable subtypes in all three systems.

The overall design of our current study is shown in Figure 1. Gastric adenocarcinomas share a variety of molecular and cellular characteristics with CRC. To assess whether CMS classification could be applied to GAC, we obtained transcriptomes from TCGA and ACRG, removed low-expression genes, and called CMS classifications using the *CMSCaller* algorithm (Figure 2A). Principal component analysis (PCA) demonstrated that TCGA and ACRG transcriptomes clustered discretely and comparably with CRC based on CMS classification (Figure 2B). Batch correction based on tumor type alone resulted in indistinguishable CMS classifications for the TCGA GAC and CRC RNA-Seq datasets derived from tumors processed using identical methods (Figure 2C).

### 3.2. CMS4 Gastric Adenocarcinomas Are an EMT-Predominant Subtype

Among the subtypes, the EMT-associated CMS4 classification was the predominant subtype in both datasets (38.8–44.3%, Figure 2D) and most prevalent in tumors of the cardia (48.5%) and antrum (46.2%) (Figure S1A). As previously reported for CRC, more advanced tumors were significantly more likely to be classified as CMS4 (*p* < 0.001, pT, Table S5) and strongly associated with miR-200 EMT inhibitor downregulation (Figure 2E), EMT pathway activation (Figure 2F, Figure S1B), and angiogenesis (Figure S1C). CMS4 tumors were nearly mutually inclusive with the ACRG EMT classification (Figure 2G) and represented a significant portion of GS, EBV, and MSS tumor subtypes (Figure 2H,I).

### 3.3. CMS1 Classifies Hypermutant Tumors with Strong Contingency for Immunotherapy Biomarkers

**Figure 3:**
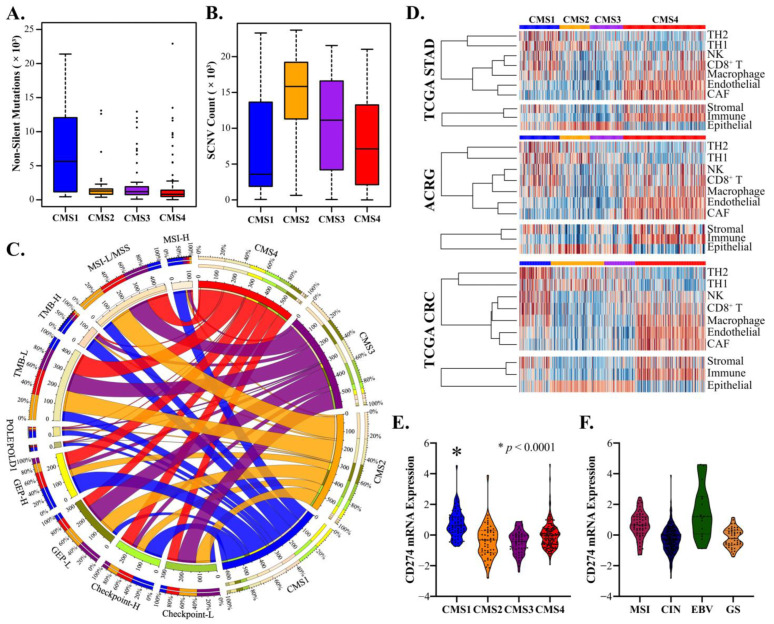
CMS1 classification is linked to immunotherapy susceptibility markers in GAC. (**A**,**B**). CMS1 demonstrated hypermutation compared to CMS2, CMS3, or CMS4 (*p* < 0.0001 for all comparisons), as well low somatic copy variation (SCNV) consistent with CMS1 in CRC. (**C**). Circos diagram demonstrating strong contingency between CMS1 in GAC and clinical markers of immunotherapy response: MSI-H, TMB, GEP, POLE/POLD1, and checkpoint. (**D**). Tumor microenvironment and immune markers from the TIMER/Cistrome and ESTIMATE projects relative to GAC CMS type, demonstrating enrichment of CD8+ T cells, NK cells, and Th cells in CMS1 and enrichment of stroma, endothelia, CAF, and macrophage markers in CMS4. (**E**,**F**). Violin plot demonstrating enriched expression of CD274 (PD-L1) in CMS1 compared to CMS2-4 (*p* < 0.0001 for all comparisons), with increased association compared to standard clinical subtypes of GAC.

The next most frequent classification was CMS1 (20.9–21.5%, Figure 2D), which was more prevalent in females than males (27.5% and 18.0%, respectively) and most frequent in the fundus region (Figure S1A,D). As in CRC, CMS1 tumors were characterized by hypermutation and low SCNV (Figure 3A,B), and GSEA identified DNA repair and unfolded protein response as key enriched pathways (Figure S1E,F). CMS1 tumors strongly overlapped with the TCGA and ACRG subtypes and were frequently classified as MSI-H (>60% for all comparisons, Figure 2G–I). Classification based on high tumor mutational burden (TMB) and microsatellite instability is a subject of intense interest due to clinical trials demonstrating sustained and durable responses to anti-PD1 therapies, such as pembrolizumab, which has led to its recent approval as a first-line therapy for MSI-H/dMMR CRC and for unresectable and metastatic TMB-high solid tumors [46]. Comparative analysis of CMS1 with accepted and investigational biomarkers for immunotherapy response demonstrated strong contingency for POLE/POLD1 (~65%), MSI-H (>60%), TMB-H (>60%), GEP-H (>40%), and Checkpoint H (~40%) (Figure 3C). Notably, analysis of TME gene markers showed mixed epithelial and stromal characteristics and a clear increase in Th_1/2_, NK, and cytotoxic T cells, mirroring results in CRC (Figure 3D). Comparatively, CMS2 and CMS3 tumors demonstrated potential enrichment of Th_1_ cells, and CMS4 tumors demonstrated mixed results for antitumor NK and CD8^+^ T cells and strong enrichment for macrophages, cancer-associated fibroblasts (CAFs), and endothelia (Figure 3D). Finally, the CMS1 classification showed strong upregulation of the CD274 gene encoding the key immunotherapy biomarker PD-L1 (Figure 3E) and was a more consistent predictor compared to the TCGA subtype (Figure 3F). These results demonstrate a specific association between the CMS1 subtype and key markers and molecular characteristics of the antitumor immune response.

### 3.4. CMS2 and CMS3 Classifications Recapitulate Their Corresponding CRC Subtypes

The canonical WNT-associated CMS2 subtype was the least prevalent in GAC and more frequent in the antrum region closer to the duodenum (Figure S1A). Assessment of the mutational landscape revealed that >95% of CMS2 tumors demonstrate APC mutation or CN loss, and unlike other GAC subtypes, there was a virtual lack of ARID1A mutations (Figure S2A). Accordingly, the expression of the WNT-regulated CSC markers LGR5 and ASCL2 was dramatically upregulated, and WNT signaling, the WNT downstream target MYC pathway, and G2/M proliferative markers were all enriched (Figure S2B–G). Moreover, strong activation of the WNT/MYC-associated miR-17/92 oncomiR cluster was apparent (Figure S2H).

CMS3 tumors are characterized by overactive metabolism and mutations in the KRAS pathway. Within the CMS3 GAC subtype, ARID1A, PI3K, KRAS, and SMAD4 mutations were more frequent (Figure S2A). GSEA showed CMS3-specific enrichment for a variety of metabolic pathways in GAC, with the exception of pyrimidine metabolism, which was enriched in CMS1 tumors, likely due to overactive DNA repair mechanisms (Figures S1E and S3A). Additionally, oxidative phosphorylation and protein secretion were significantly enhanced in this subtype (Figure S3B,C). Taken together, these findings show that CMS2 and CMS3 classifications recapitulate their associated characteristics associated in CRC.

### 3.5. CMS4 Is an Independent Predictor of GAC Overall Survival

**Figure 4:**
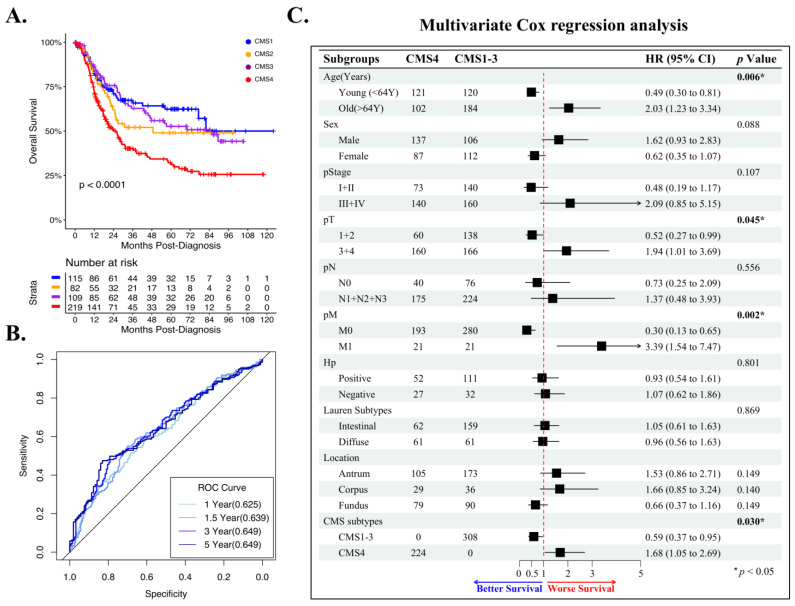
CMS4 is an independent predictor of GAC overall survival. (**A**) CMS4 has the poorest overall survival of any CMS classification in the combined ACRG/STAD dataset, with a median OS of approximately 24.2 months, compared to 48.7 months for CMS2, 85.6 months for CMS3, and >120 months for CMS1 (*p* < 0.0001). (**B**) ROC analysis demonstrating moderate sensitivity and specificity for OS in the combined ACRG/STAD dataset at 1, 1.5, 3, and 5 years postdiagnosis (AUC: 0.625–0.649, *p* < 0.0001). (**C**) Multivariate Cox regression analysis demonstrating that CMS4 is an independent prognostic factor for OS in GAC (*p* = 0.030).

To assess whether CMS classification could predict outcomes in GAC, we performed univariate overall survival (OS) analysis using the combined TCGA/ACRG datasets. CMS4 predicted significantly reduced OS in GAC patients when compared to CMS1-3 classifications (Figure 4A, *p* < 0.0001). Among CMS classifications, CMS1 showed the best survival, whereas CMS2-3 showed intermediate survival (Figure 4A, Table S1). Notably, CMS classification appeared to be a better predictor of OS compared to traditional TCGA classifications (Figure S3D). To further assess the predictive ability of CMS4 in GAC, we performed an ROC analysis and found areas under the curve (AUCs) of 0.625, 0.639, 0.649, and 0.649 for 1, 1.5, 3, and 5 years postdiagnosis, respectively (Figure 4B). CMS4 is also a predictor of outcomes in CRC but is strongly associated with advanced stage [9]. To control for the potentially confounding effects of stage and other significant clinical factors identified using contingency analysis (Table S5), we performed a multivariate Cox regression analysis. Among the factors, tumor size (pT), distant metastasis (pM), age, and CMS4 were significant and independent prognostic factors of patient OS (Figure 4C).

### 3.6. CMS1 Is a Favorable Response Indicator for Pembrolizumab in Metastatic GAC

**Figure 5:**
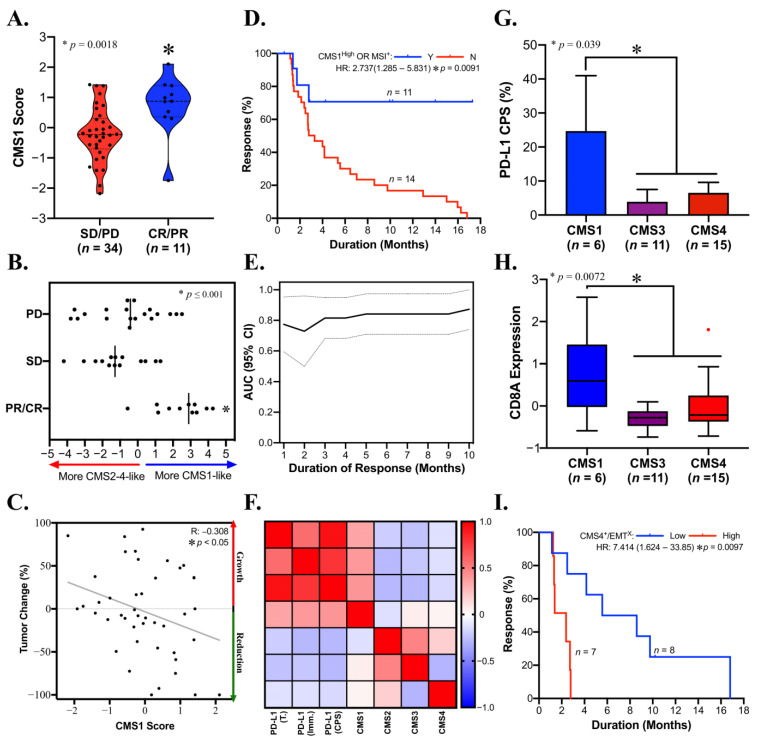
CMS1 predicts the response to subsequent lines of anti-PD1 (pembrolizumab) in advanced GAC. (**A**) CMS1 score is significantly increased in pembrolizumab responders in GAC (*p* = 0.0018). (**B**) CMS1^High^/CMS2-4^Low^ patients have improved responses to pembrolizumab (*p* ≤ 0.001). (**C**) CMS1 score is linearly correlated with a reduction in tumor burden (*p* < 0.05, R = −0.308). (**D**) CMS1 combined with standard dMMR-based MSI status (n = 9 and n = 3 respectively) predicts sustained durability of pembrolizumab therapy. (**E**) ROC analysis from 1–14 months post treatment shows that the CMS1 score is sensitive and specific for prediction of pembrolizumab response at 9, 10, and 13 months post treatment (AUC: 0.7179–0.7833, *p* < 0.05). (**F**) Correlation matrix demonstrating that the CMS1 score is positively correlated with PD-L1 immunohistochemistry scoring, whereas CMS2-4 scores are not. (**G**). PD-L1 CPS is significantly upregulated in CMS4 classified as advanced GACs compared to CMS3/4 (*p* = 0.0309). (**H**) The cytotoxic T-cell marker CD8A is significantly upregulated in CMS1 classified as advanced GACs compared to CMS3/4 (*p* = 0.0072). (**I**). CMS4/EMT^Low^ tumors demonstrated a significantly longer duration of response to pembrolizumab compared to CMS4/EMT^High^ tumors (median PFS: 7.079 and 2.378 months, respectively; *p* = 0.0097).

Anti-PD1/PD-L1 therapies are emerging as a key tool against intractable solid tumors, and biomarkers to identify responders are in demand. CMS1 is favorably associated with a variety of approved immunotherapy biomarkers, including TMB, GEP, and MSI (Figure 3C). To ascertain the practical significance of CMS1 as an immunotherapy biomarker, we sought to assess outcome measures in metastatic GAC patients treated with anti-PD1 (pembrolizumab). The CMS1 signature was significantly elevated in patients with partial or complete responses to pembrolizumab (*p* < 0.002, Figure 5A). To assess this factor in tumors that may have mixed CMS characteristics, we calculated a signature based on all four CMS types. Patients with more CMS1-like tumors in this context were also significantly more likely to respond to pembrolizumab (*p* ≤ 0.0001; Figure 5B). Moreover, CMS1 correlated with a reduction in tumor burden after pembrolizumab therapy (*p* < 0.05, R: −0.308; Figure 5C), and CMS1 classification identified some responders independent of MSI status (data not shown). In support of these findings, patients with CMS1^High^ classification or MSI had a significantly increased duration of response to pembrolizumab (*p* = 0.0091, Figure 5D), and CMS1^High^ status was able to detect responders by ROC analysis for 9, 10, and 13 months post pembrolizumab, with AUCs ranging from 0.7179 to 0.7833 (*p* < 0.05; Figure 5E, Figure S4). PD-L1 IHC is a biomarker for pembrolizumab response. Among CMS classifications, only CMS1 was positively correlated with this marker (Figure 5F). Furthermore, PD-L1 CPS was significantly upregulated in CMS1 compared to CMS3-4 tumors (*p* = 0.0309; Figure 5G), which was accompanied by upregulation of the cytotoxic T-cell marker CD8A (*p* = 0.0072; Figure 5H). Recent studies suggest that solid tumors with EMT/TGF-beta signaling characteristics may be reconditioned for anti-PD1 immunotherapy by targeting these factors [47]. Analysis of CMS4 tumors with low or high levels of EMT suggested that this may be the case, with CMS4/EMT low tumors demonstrating a significantly increased duration of response to pembrolizumab (*p* = 0.0097, Figure 5I). 

### 3.7. CMS Classification Significantly Overlaps with Other GAC Molecular Subtypes

**Figure 6:**
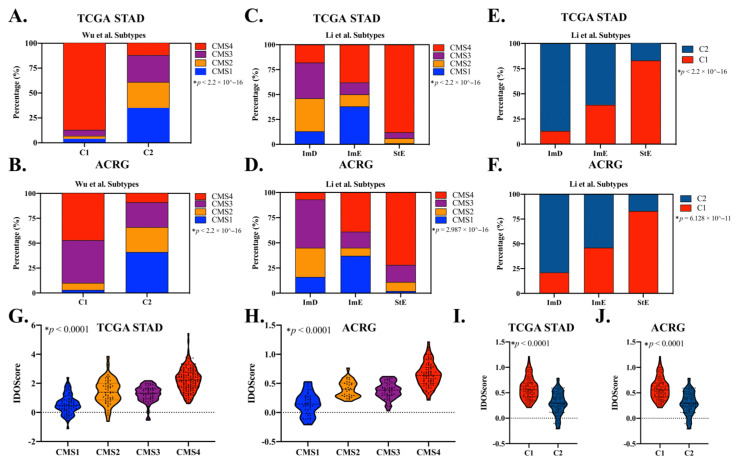
The CMS subtyping system has meaningful overlap with other immune signature-based classifications in GAC. (**A**,**B**) CMS proportion in Wu et al. molecular subtypes. Proportions of 88% and 48% of C1 subtype were represented as CMS4, whereas 35% and 41% of C2 subtypes were represented as CMS1 in TCGA-STAD (**A**) and ACRG (**B**) cohorts, respectively. (**C**,**D**) CMS proportion in Li et al. molecular subtypes. Proportion of 88% and 72% of StE were represented as CMS4, and 69% and 77% of ImD were represented as CMS2+CMS3 in TCGA-STAD (**C**) and ACRG (**D**) cohorts, respectively. ImE was represented as an equal proportion of CMS1 (37–38%) and CMS4 (38–39%) in both GAC cohorts. (**E**,**F**) Wu et al. subtype proportions among Li subtypes. A proportion of 83% of StE were represented as C1 subtype, and ImE was represented as a nearly equal portion of C1 and C2 in both TCGA-STAD (**E**) and ACRG (**F**) cohorts. Proportion of 87% and 79% of ImD were represented as C2 subtypes in TCGA-STAD and ACRG cohorts, respectively. (**G**–**J**). IDOScore in each subtyping system. CMS4 had the highest score, whereas CMS1 had the lowest IDOScore among all CMS subtypes both in TCGA-STAD (**G**) and ACRG (**H**) cohorts. Wu et al. C1 subtype had a higher IDOSocre than C2 subtype both in TCGA-STAD (**I**) and ACRG (**J**) cohorts. Fisher’s exact test was performed for (**A**–**F**), Kruskal–Wallis test was performed for (**G**,**H**), and Wilcoxon test was performed for (**I**,**J**); * *p* < 0.0001 for all comparisons.

Recently, Wu et al. and Li et al. reported molecular subtypes based on immune signatures and pathway activities associated with immunity, DNA repair activity, oncogenic signaling, and stromal signatures [48,49]. Both groups of researchers addressed the effect of intratumor heterogeneity on immune activity, stromal signatures, and clinical outcomes in GAC [48,49]. We found that the CMS classification system had meaningful overlap with both systems. Notably, CMS4 overlapped significantly with the C1 subtype developed by Wu et al., which is characterized by resting immune activity, EMT, angiogenesis, and worse clinical outcomes. Furthermore, most CMS1 GACs fell under the category of the C2 subtype, which is characterized by increased immune activity and improved overall survival in both in TCGA-STAD and ACRG cohorts (*p* < 0.0001; Figure 6A,B). Despite these similarities, the C1/2 subtype showed no difference in terms of response to anti-PD-1 therapy (median PFS: 2.73 and 4.08 months, respectively; *p* = 0.69 and *p* = 0.44, respectively; Figure S5A,B). Similarly, CMS4 overlapped significantly with the StE subtype established by Li et al., which is characterized by strong stromal signatures, including TGF-β pathway activation, a high level of antitumor immunity, and worse prognosis both in TCGA-STAD and ACRG cohorts (*p* < 0.0001; Figure 6C,D). CMS2 plus CMS3 overlapped with the ImD subtype of this system, which is characterized by low immune infiltration, high DNA damage repair activity, high tumor aneuploidy and intra-tumor heterogeneity, and frequent TP53 mutations (*p* < 0.0001; Figure 6C,D). Most of the CMS1 subtype overlapped with the ImE subtype of this system, which is characterized by strong immune infiltration and favorable prognosis both in TCGA-STAD and ACRG cohorts (*p* < 0.0001; Figure 6C,D). However, the ImE subtype had equal proportions of CMS1 (37–38%) and CMS4 (38–39%) (Figure 6C,D). Only 3 of 45 pembrolizumab-treated GAC patients were classified as StE subtype using this system (data not shown). Therefore, it was not possible to compare the response rate. Unsurprisingly, there were also notable overlaps between the C1/C2 system developed by Wu et al. and the Li et al. subtypes (Figure 6E,F).

In addition to the molecular subtypes, Li et al. established a pathway-based prognostic factor and immunotherapy response indicator relying on the expression of four genes (TAP2, SERPINB5, LTBP1, and LAMC1), termed IDOScore, which is an adverse prognostic factor and inversely related to the immunotherapy response [49]. CMS4 and CMS1 showed the highest and lowest IDOScores, respectively, among CMS subtypes in TCGA-STAD and ACRG cohorts (*p* < 0.0001; Figure 6G,H). Similarly, Wu et al.’s C1 subtype, which has significant overlap with CMS4, had a higher IDOScore compared with the C2 subtype both in TCGA-STAD and ACRG cohorts (*p* < 0.0001; Figure 6I,J). GACs with complete or partial response to pembrolizumab tended toward a higher IDOScore than those with progressive or stable disease (*p* = 0.0083, Figure S5C). However, IDOScore was not predictive of response duration (median PFS: 3.99 and 2.78 months, respectively; *p* =0.093; Figure S5D).

## 4. Discussion

Previous classification systems described for GAC include the TCGA (EBV, MSI-H, GS, and CIN) [47] and the ACRG (MSI, MSS/TP53^WT^, MSS/TP53^MUT^, and EMT) [39] subtypes. Although both systems have various potential prognostic uses, neither has gained clinical prominence. This is perhaps due to limited clinical studies and data to predict therapeutic response. The CMS classification system is now being used to reassess past clinical trials and perform prospective trials in CRC. A variety of research findings have linked CMS to important clinical characteristics that may be applicable to both CRC and GAC, including the response to targeted therapy and antitumor immunity. Importantly, a variety of platforms have been developed to assess CMS in a cost-effective manner, ranging from less economical gene expression signatures to highly economical neural convolutional networks that can predict CMS status from simple H&E images [50].

In this analysis, we demonstrated that CMS classification can be effectively applied to GAC and that each CMS subtype essentially recapitulates the corresponding CRC subtype. CMS4 was the predominant subtype in GAC, characterized by EMT activation, stromal invasion, angiogenesis, and worsened clinical outcomes. CMS1 was the second most frequent subtype and showed specific association with key markers and molecular characteristics of antitumor immune response, hypermutation, low SCNV, immune activation, and the best clinical outcomes. CMS3 was the third most frequent subtype and characterized by overactive metabolism, KRAS mutation, and intermediate outcomes. CMS2 was the least frequent subtype and featured WNT and MYC activation, high epithelial activity, APC mutation, lack of ARID1A, and intermediate outcomes.

CMS4 is the most common classification in GAC and more likely to be present in patients diagnosed in an advanced stage of disease. Recently, the use of the anti-PD1 monoclonal antibody pembrolizumab has been expanded in GI cancers, including GAC. Pembrolizumab is approved as a subsequent line of therapy in GAC patients with tumors demonstrating dMMR/MSI or PD-L1 positivity and in combination with trastuzumab and chemotherapy for the first-line treatment of patients with locally advanced unresectable or metastatic HER2-positive GAC [31,32,33]. In the current study, CMS4 demonstrated mixed results for antitumor immune response and strong EMT activation, and CMS4 status did not predict a favorable response to pembrolizumab. However, stratifying CMS4 tumors based on their relative EMT status revealed a longer duration of response in CMS4/EMT^Low^ patients (median PFS 7.097, *p* = 0.0097). These findings suggest the interesting possibility that CMS4 tumors could be reconditioned to respond to anti-PD1/PD-L1 therapies via coadministration of TGFβ inhibitors, as recently suggested [45,47].

MSI/dMMR status is an incomplete predictor of the response to anti-PD1 immunotherapy in GAC. Clinically, additional diagnostics, including TMB and PD-L1 IHC, are also useful but limited. Clinical studies show that EBV status is an excellent predictor of immunotherapy response but is often impractical to test and not commonly implemented in clinical care. CMS classification is backed by a variety of studies in CRC, and clinically useful biomarkers have been developed based on it, which could be applied to GAC. Our findings demonstrate that CMS1 status is a predictor of the response to pembrolizumab. Moreover, we showed that combining CMS1 status with MSI, as determined by clinically practical dMMR IHC, leads to improved identification of patients with durable responses to pembrolizumab. Overall, the current study demonstrates that CMS1 and CMS4/EMT low tumors may be excellent targets for approved anti-PD1 therapy pembrolizumab and that identifying patients with these characteristics may enable further personalization of therapy to improve outcomes in GAC patients.

Recently, several studies identified molecular subtypes with GAC. Notably, Wu et al. reported two molecular subtypes termed C1 and C2 based on 390 immune-related genes. Furthermore, Li et al. reported three subtypes termed ImD, ImE, and StE subtypes based on 15 pathways, including immune, DNA repair activity, oncogenic, and stromal signatures, and subsequently developed IDOScore to predict clinical outcome and immunotherapy response based on the mRNA expression of four genes (TAP2, SERPINB5, LTBP1, and LAMC1). To compare the CMS system with these subtypes, we performed analyses to assess subtype overlap. CMS4 had significant overlap with the C1 subtype identified by Wu et al., which is characterized by a rested immune state, EMT, and poor clinical outcomes, whereas most CMS1 GACs fell under the category of the C2 subtype, which is characterized by increased immune activity and improved overall survival [48]. Among the Li et al. subtypes, CMS4 strongly overlapped with the StE subtype, which is characterized by EMT activation, poor clinical outcomes, and reduced immunotherapy response. The combination of CMS2 and CMS3 mostly overlapped with the ImD subtype, which has low immune infiltration, high DNA repair activity, high tumor aneuploidy and intratumor heterogeneity, and frequent TP53 mutations. Furthermore, CMS1 overlapped meaningfully with the ImE subtype, which is characterized by elevated immune activation, high tumor mutational burden, microsatellite instability, and increased PD-L1 expression [49]. Both CMS1 and CMS4 equally contributed to the ImE subtype in the Li et al. system [49]. Additionally, CMS1 and CMS4 had the lowest and highest measures for IDOScore, respectively, an adverse prognostic factor that was inversely related to the immunotherapy response in the Li et al. study [49]. These comparisons demonstrate that the molecular characteristics among these subtyping systems have notable similarities. However, CMS classification adds to our knowledge of the existing experimental subtypes and has some specific advantages. First, CMS classification can further divide the C2 subtype, which may be useful in studies focused on this classification. Additionally, it can robustly predict the response to pembrolizumab, which is not possible for C1/2 subtypes and difficult to assess because of a limited amount of StE subtypes using current limited datasets. Li et al.’s established subtyping system also depends on transcriptomic sequencing, although it may not be able to distinguish between patients in smaller cohorts. For instance, in the cohort of pembrolizumab-treated patients used in this study, nearly all patients (35/45) fell under the ImE category. Comparatively, we were able to predict patient response (duration) to pembrolizumab by assessing CMS4-categorized tumors for their EMT activation status and found that CMS4/EMT-low patients had a significantly expanded duration of response (median PFS: 7.079; *p* = 0.0097). Another notable advantage of CMS classification is that due to its extensive use in CRC, there have been a variety of technological developments that can be fine-tuned and applied to GAC, for example, in CRC, machine-learning algorithms that can assign accurate CMS classification from whole-slide H&E staining [50] and a CMS assay developed and validated for clinical use with formalin-fixed, paraffin-embedded (FFPE) samples [51].

## 5. Conclusions

**Figure 7:**
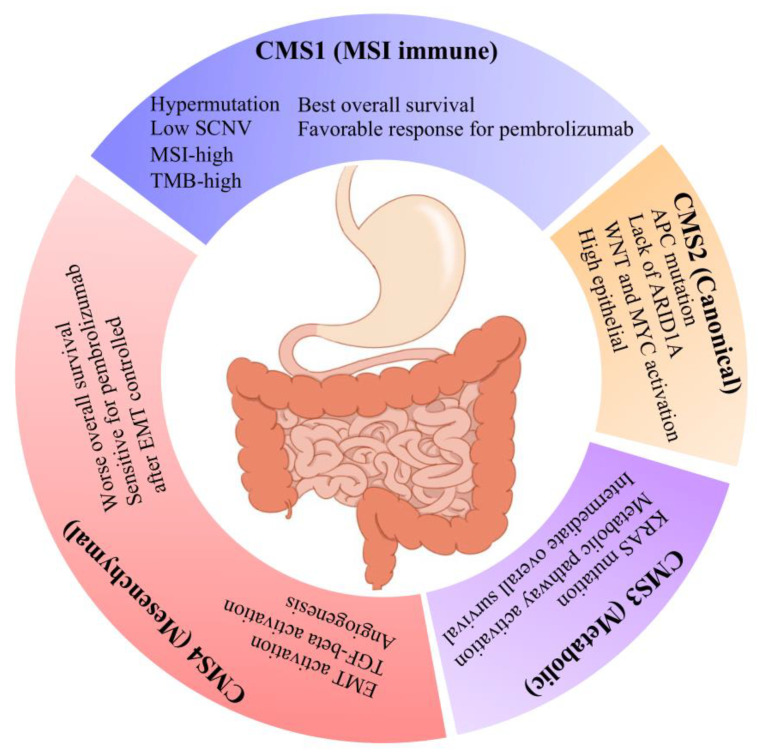
Key characteristics of GAC CMS subtypes. This graphic demonstrates some of the predominant features associated with each of four CMS subtypes of GAC, which are similar to CRC.

In summary, our findings demonstrate that CMS classification can be applied to GAC and that the resulting subtypes strongly overlap with their CRC counterparts. Among these subtypes, the EMT-related CMS4 classification is an independent predictor of GAC outcomes. Importantly, in metastatic GAC patients receiving subsequent courses of pembrolizumab immunotherapy, the CMS1 classifier can predict both reductions in tumor burden and durability of response, and patients in the CMS4 subtype may be re-sensitized to immunotherapies such as pembrolizumab after controlling for the influence of EMT. In conclusion, our study highlights an important topic for GAC management in the post-immunotherapy era (Figure 7).

## Figures and Tables

**Table 1:** Baseline characteristics.

	TCGA-STAD (*n* = 415)	ACRG (*n* = 300)	TCGA-CRC (*n* = 380)	Pembrolizumab-Treated GAC (*n* = 45)
Age(year)	65 (30–90)	62 (24–86)	65 (30–90)	57 (26–78)
Gender				
Male	268 (64.6%)	199 (66.3%)	207 (54.5%)	32 (71.2%)
Female	147 (35.4%)	101 (33.7%)	169 (44.5%)	13 (28.9%)
pStage				
I	57 (13.7%)	30 (10%)	57 (15%)	0 (0%)
II	123 (29.6)	96 (32%)	118 (31.1)	0 (0%)
III	171 (41.2%)	95 (31.7%)	113 (29.7%)	0 (0%)
IV	41 (9.9%)	77 (25.7%)	52 (13.7%)	45 (100%)
Missing	23 (5.5%)	2 (0.6%)	40 (10.5%)	0 (0%)
Primary tumor site				
Antrum/Distal	156 (37.6%)	155 (51.7%)	NA	NA
Cardia/GEJ	97 (23.4%)	32 (10.7%)	NA	NA
Fundus/Body	143 (34.5%)	107 (35.7%)	NA	NA
Lauren type				
Intestinal		150 (50%)	NA	NA
Diffuse		142 (47.3%)	NA	NA
OS/PFS (year)	1.57 (0–10.2)	4.2 (0.08–8.8)	2.6 (0–12.3)	0.5 (0.05–1.4)
Pembrolizumab Response				
CR/PR	NA	NA	NA	11 (24.4%)
SD/PD	NA	NA	NA	34 (75.6%)

GEJ: gastroesophageal junction; OS: overall survival; PFS: progression-free survival; CR: complete response; PR: partial response; SD: stable disease; PD: progressive disease.

## Data Availability

All data used in this study are already publicly available. Paired-end FASTQ data for GAC patients treated with the programmed cell death 1 (PD-1) inhibitor pembrolizumab (n = 45) were obtained from the European Nucleotide Archive (ENA: PRJEB25780). Copy number profile, RNA-seq, DNA methylation, and miRNA-seq data from TCGA gastric adenocarcinoma (STAD) and colorectal cancer (CRC) datasets were obtained from the University of California, Santa Cruz (UCSC), Cancer Genome Browser (https://xenabrowser.net) [37]. The expression profiling of the Asian Cancer Research Group (ACRG) study of gastric tumors (GSE62254; n = 300) was downloaded from the NCBI Gene Expression Omnibus (GEO).

## Supplementary Materials

The following supporting information can be downloaded at: https://www.mdpi.com/article/10.3390/cancers14153740/s1, Figure S1: A. CMS4 and CMS1 was most prevalent in tumors of cardia, antrum and fundus. CMS4 was strongly associated with EMT activation (B) and angiogenesis (C) (D) CMS1 was more frequent in females than males. CMS1 was significantly associated with DNA repair (E) and unfolding protein response (F). Figure S2. A. The mutation landscape of TCGA-STAD based on CMS subtypes. Cancer stem cell markers LGR5 (B) and ASCL2 (C) were significantly upregulated in CMS2. Wnt signaling pathway (D) and its downstream target MYC pathway (E,F), as well as G2/M checkpoint (G), were enriched in CMS2 and CMS3. H. WNT/MYC-associated miR-17/92 oncomiR clusters were strongly associated with CMS2. Figure S3. A. GSEA analysis of gastric adenocarcinoma based on CMS in TCGA and ACGR. Oxidative phosphorylation (B) and protein secretion (C) were enriched in CMS3. D. MSI has the best overall survival of all clinical subtypes in the combined ACRG/TCGA-STAD dataset, with a median OS of approximately 81.8 months compared to 27.7 months for CIN, 61.63 months for EBV, and 42.9 months for GS (*p* = 0.036). Figure S4. ROC curve for CMS1 score to predict anti-PD-1 response at 9, 10, and 13 months after pembrolizumab treatment. Figure S5. Wu et al. and Li et al. molecular subtyping systems do not predict the anti-PD-1 response in a limited cohort. A. In the pembrolizumab-treated GAC cohort, 25% and 75% of CR/PR patients were C1 and C2 subtype, as reported by Wu et al., respectively, and 38% and 62% of SD/PD patients were C1 and C2 subtype (*p* = 0.69), respectively. B. C1 and C2 subtypes had similar duration of response to pembrolizumab (median PFS 2.73 and 4.08 months, respectively; *p* = 0.44). C. Patients with complete or partial response to pembrolizumab-treated GAC had a lower IDOScore than those with stable or progressive disease (median IDOScore of 1244 and 2464, respectively; *p* = 0.0083). D. Lower IDOScore (<median 1919) patients had similar duration of response to pembrolizumab compared with higher IDOScore patients (median PFS 3.99 and 2.78 months, respectively; *p* = 0.093). Table S1. Clinical patient characteristics for stomach cancer. Table S2. ESTIMATES analysis for STAD, ACRG, and CRC. Table S3. Estimation of EMT activation of STAD from TCGA. Table S4. Enrichment scores for gene set mRNA enrichment analysis. Table S5. Fisher’s exact test for STAD and ACRG.
