# Supplementary material for: Consensus Molecular Subtypes Efficiently Classify Gastric Adenocarcinomas and Predict the Response to Anti-PD-1 Immunotherapy
Source: Cancers (Basel). 2022 Jul 31;14(15):3740. doi: 10.3390/cancers14153740 (PMC9367605; doi:10.3390/cancers14153740)
Supplement: Supplementary file 1 [file cancers-14-03740-s001.zip › cancers-1753514-supplementary/Revised supplementary/Wu et al revised supplementary data.pdf]

Consensus molecular subtypes efficiently classify gastric adenocarcinomas and predict the response to anti-PD-1 immunotherapy

Xiangyan Wu, Yuhan Ye, Kenneth J. Vega and Jiannan Yao

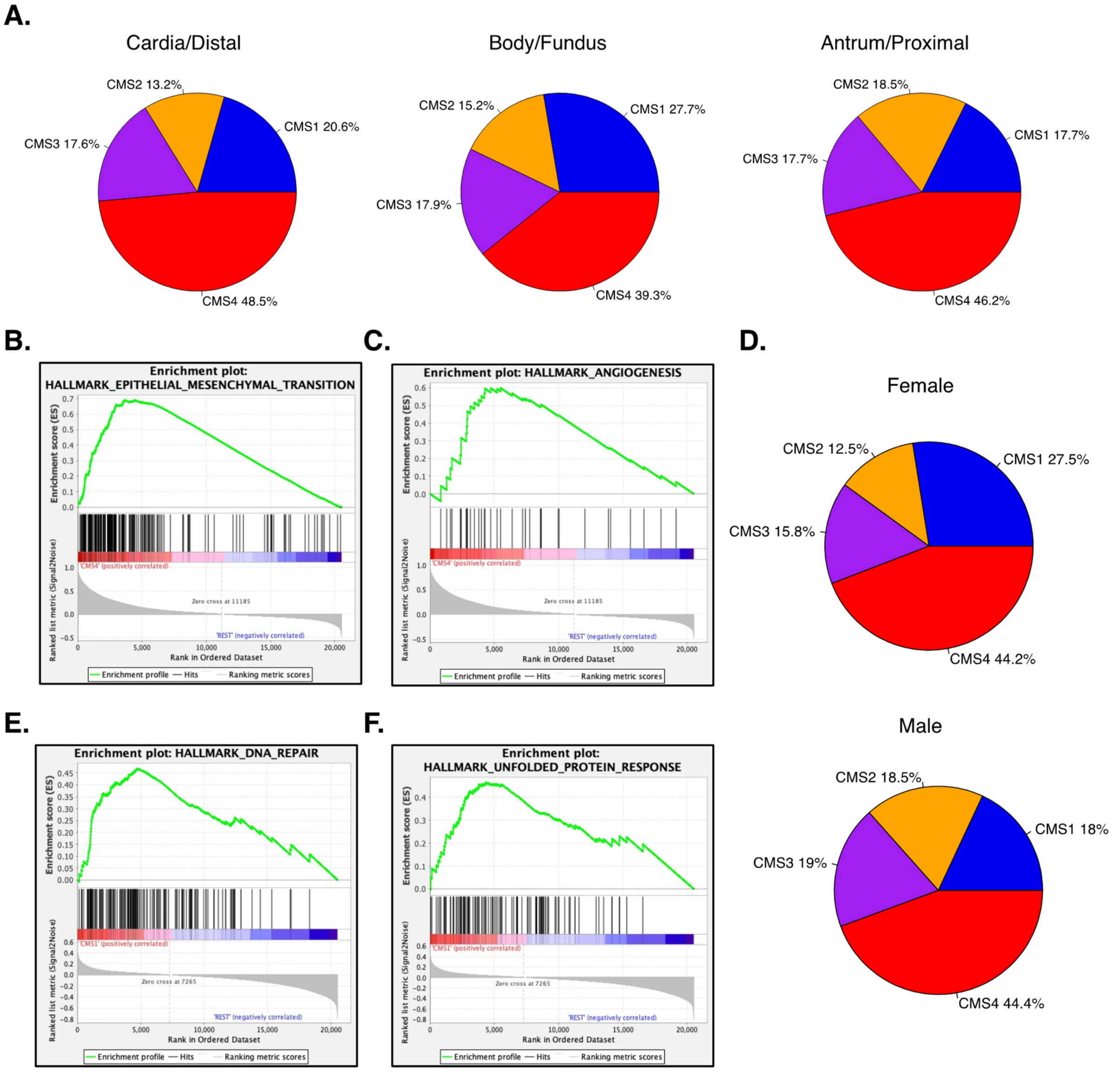

Figure S1. A. CMS4 and CMS1 was most prevalent in tumor of Cardia and Antrum and Fundus respectively. CMS4 was strongly associated with EMT activation **(B)** and angiogenesis **(C)**. **D.** CMS1 was more frequent in females than males. CMS1 was significantly associated with DNA repair **(E)** and unfold protein response **(F).**

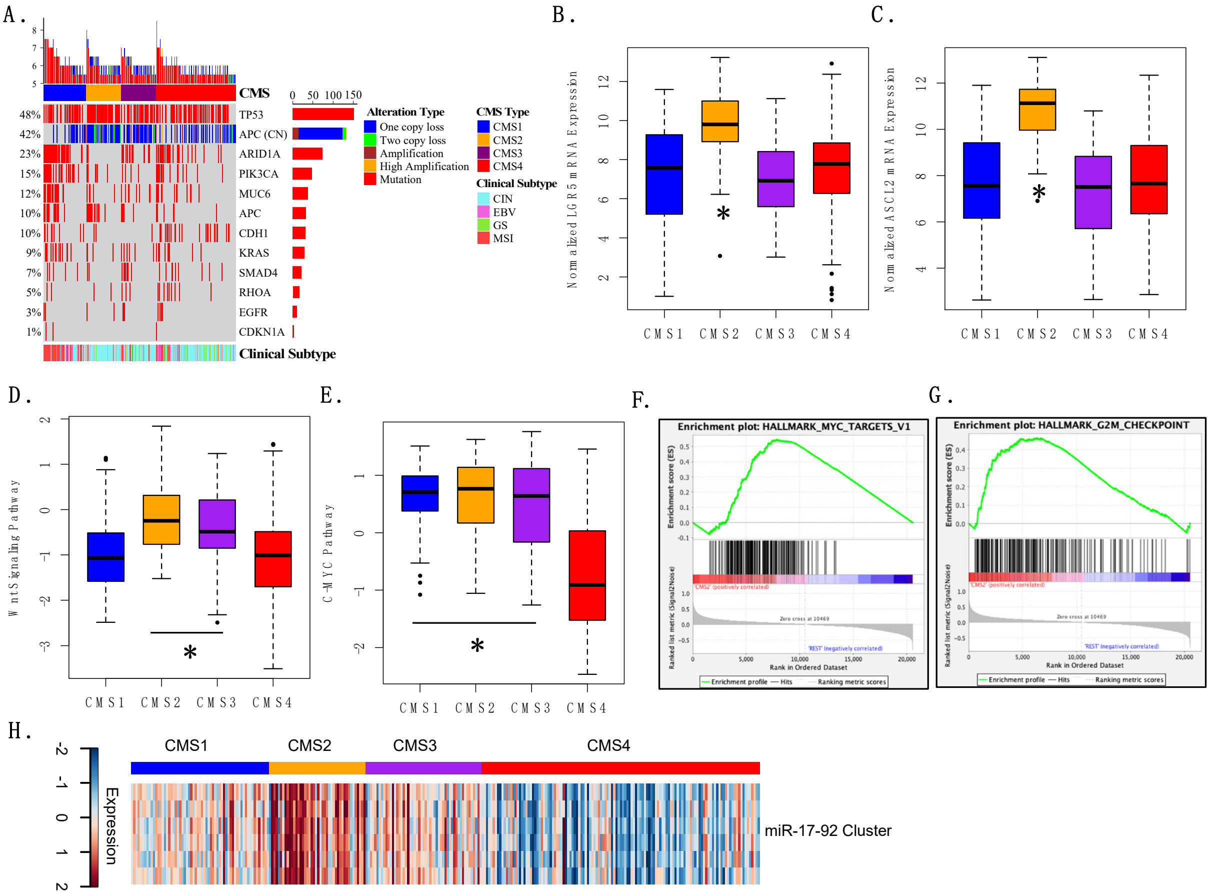

Figure S2. A. The mutation landscape of TCGA-STAD based on CMS subtypes. Cancer stem cell markers LGR5 **(B)** and ASCL2 **(C)** were significantly upregulated in CMS2. Wnt signaling pathway **(D)** and its downstream target MYC pathway **(E, F)** as well as G2/M checkpoint **(G)** were enriched in CMS2 and CMS3. **H.** WNT/MYC associated miR-17/92 oncomiR cluster were strongly associated with CMS2.

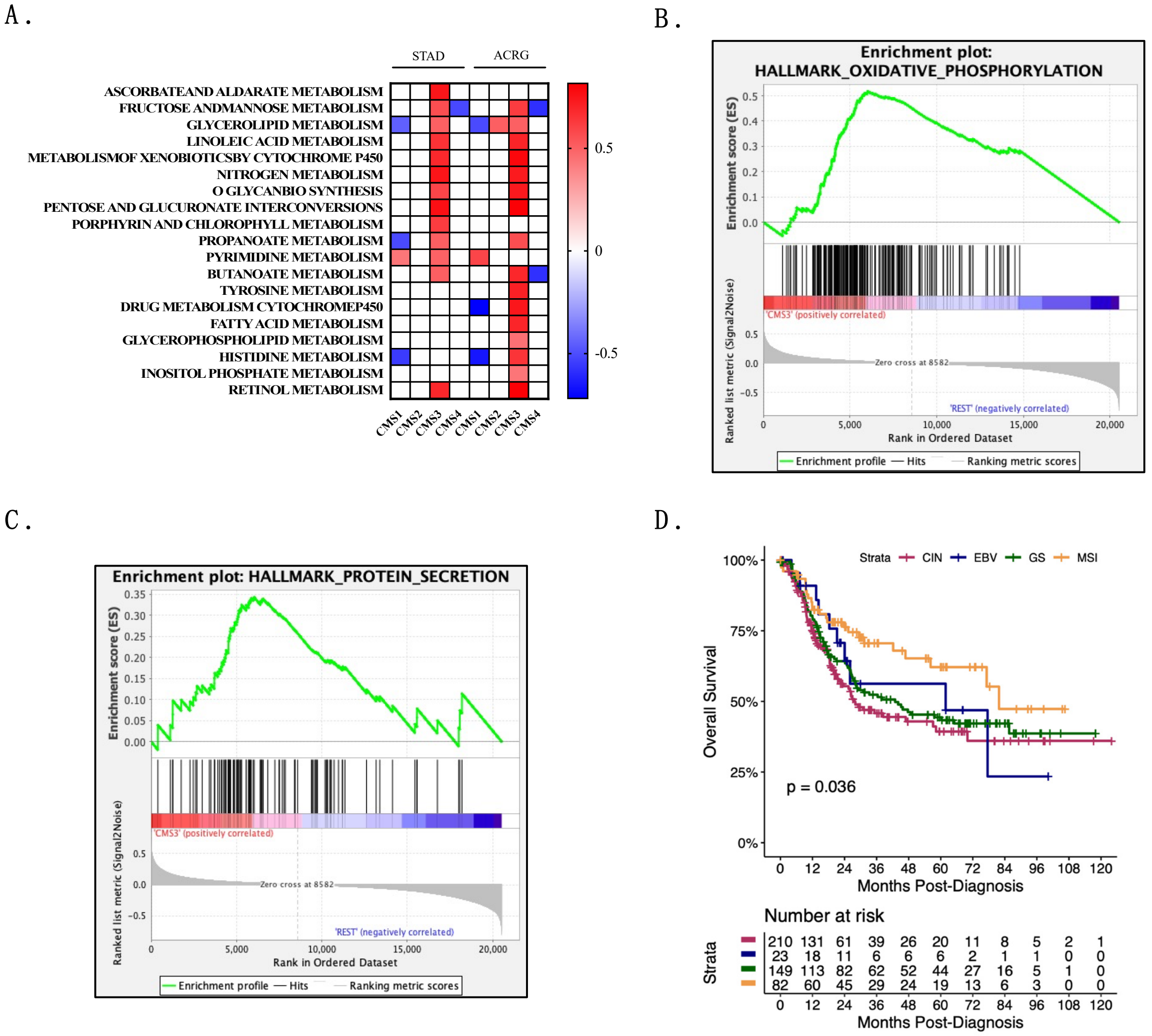

Figure S3. A. GSEA analysis of gastric adenocarcinoma based on CMS in TCGA and ACGR. Oxidative phosphorylation **(B)** and protein secretion **(C)** were enriched in CMS3. **D.** MSI has the best overall survival of any clinical subtypes in the combined ACRG/TCGA-STAD dataset with a median OS of approximately months compared to months for CIN, months for EBV, and months for GS (p = 0.036).

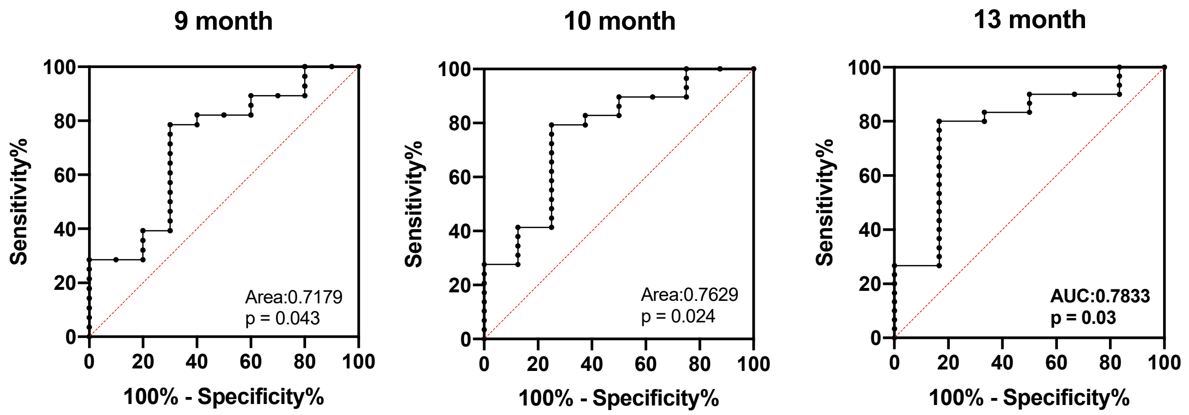

Figure S4. ROC curve for CMS1 score to predict anti-PD-1 response at 9-, 10-, and 13-month after pembrolizumab treatment.

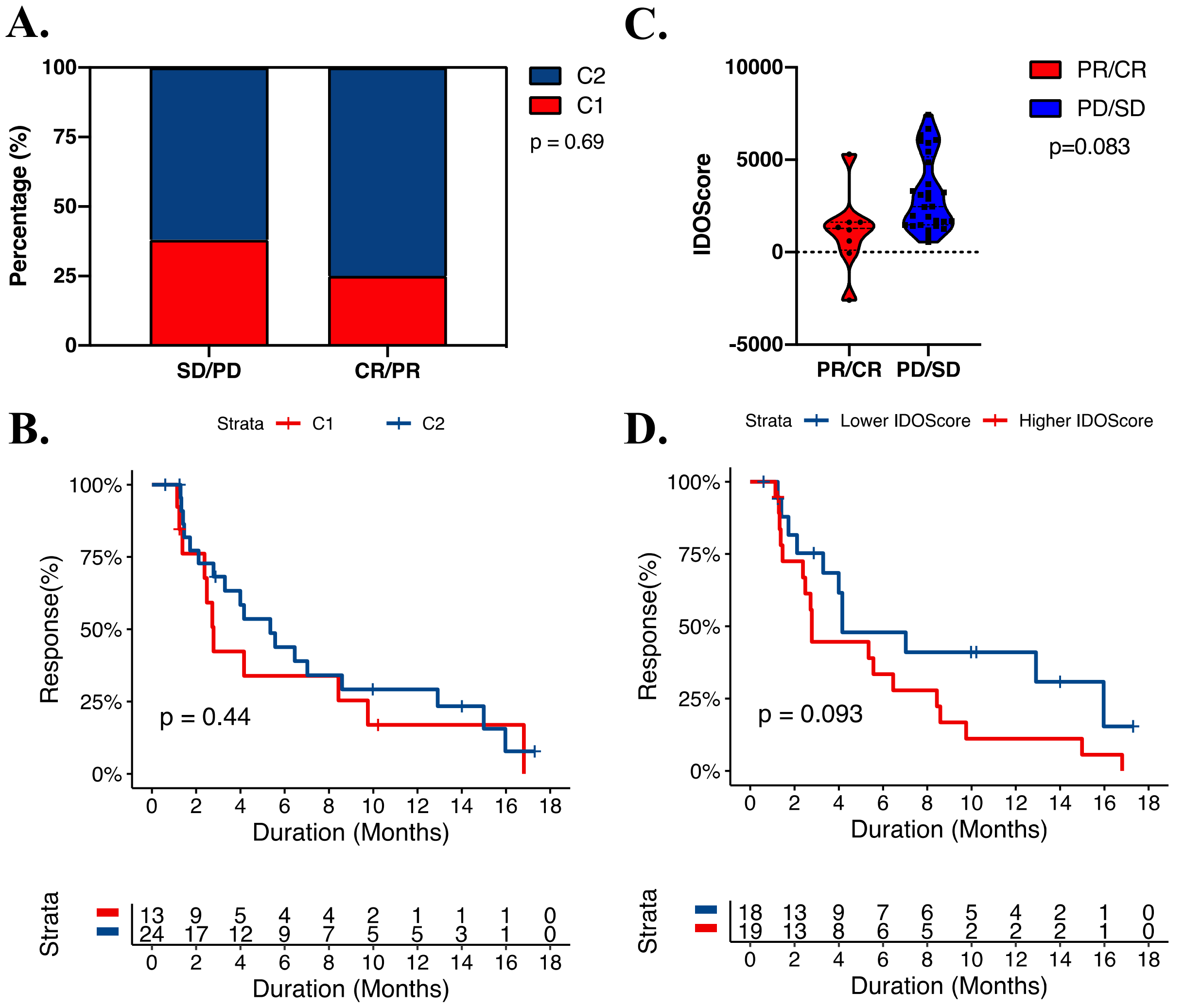

Figure S5. Wu. et al. and Li. et al. molecular subtyping systems do not predict the anti-PD-1 response in a limited cohort. A. In the pembrolizumab treated GAC cohort, 25% and 75% of CR/PR patients were C1 and C2 subtype reported by Wu. et al. respectively, and 38% and 62% of SD/PD patients were C1 and C2 subtype (p=0.69). **B.** C1 and C2 subtypes had similar duration of response to pembrolizumab (median PFS 2.73 and 4.08 months respectively, p = 0.44). **C.** Patients with complete or partial response to pembrolizumab treated GAC had a lower IDOScore than those with stable or progressive disease (median IDOScore of 1244 and 2464 respectively, p = 0.0083). **D.** Lower IDOScore (<median 1919) patients had similar duration of response to pembrolizumab compared with higher IDOScore patients (median PFS 3.99 and 2.78 months respectively, p = 0.093).

Table S1. Clinical patient characteristics for stomach cancer

Characteristics	N	HR (95%CI)	P value
**Age**			0.067
< 65	139	0.71(0.50-1.02)	
> 65	181	1.40(0.98-2.02)	
**Gender**			0.171
Male	205	1.30(0.89-1.91)	
Female	120	0.77(0.52-1.12)	
**Race**			0.463
White	209	1.19(0.75-1.88)	
Others	74	0.84(0.53-1.33)	
**pStage***			**<0.001**
I+II	124	0.47(0.31-0.69)	
III+IV	172	2.15(1.44-3.20)	
**pT**			0.026
1+2	81	0.59(0.38-0.94)	
3+4	236	1.68(1.07-2.66)	
**pN***			**0.001**
0	86	0.45(0.27-0.73)	
1+2+3	222	2.24(1.37-3.66)	
**pM***			**0.007**
M0	287	0.43(0.23-0.80)	
M1	21	2.34(1.26-4.36)	
**HP**			0.182
Positive	18	0.53(0.21-1.35)	
Negative	115	1.89(0.74-4.80)	
**Location**			
Antrum/Distal	164	1.16(0.77-1.75)	0.482
Cardia/Proximal, GE junction	45	1.21(0.74-1.97)	0.446
Fundus/Body	102	0.86(0.57-1.30)	0.482
**Lauren type**			0.001
Intestinal	122	0.30(0.15-0.61)	
Diffuse	11	3.34(1.64-6.82)	
**CMS subtype***			
CMS1	70	0.56(0.35-0.91)	**0.019**
CMS2	53	1.24(0.64-2.38)	0.525
CMS3	58	1.32(0.73-2.36)	0.360
CMS4	144	1.77(1.10-2.86)	**0.019**

*$p < 0.05$

Table S5. Fisher's exact test for STAD and ACRG

Characteristics	TCGA-STAD					ACRG				
	CMS1	CMS2	CMS3	CMS4	Fisher's Exact Test	CMS1	CMS2	CMS3	CMS4	Fisher's Exact Test
**Age**					0.061					0.218
< 65/<62	24	20	21	74		16	10	24	41	
> 65/>62	45	33	34	69		29	20	28	39	
**Gender**					0.162					0.321
Male	37	38	39	91		27	23	32	46	
Female	33	15	19	53		18	7	20	34	
**Race**					0.362					NA
White	43	31	32	103		NA	NA	NA	NA	
Others	15	17	12	30		NA	NA	NA	NA	
**pStage**					0.558					**0.002***
I+II	20	22	29	53		24	17	18	20	
III+IV	36	28	28	80		21	13	34	60	
**pT**					**0.003***					**<0.001***
1+2	20	10	25	26		33	25	25	34	
3+4	48	41	33	114		12	5	27	46	
**pN**					0.332					**0.001***
0	24	15	15	32		12	8	2	8	
1+2+3	44	33	42	103		33	22	50	72	
**pM**					0.942					0.619
M0	64	48	51	124		42	27	48	69	
M1	4	4	3	10		1	3	3	4	
**HP**					0.114					0.725
Positive	4	4	1	9		10	6	7	18	
Negative	30	19	32	34		11	6	13	18	
**Location**					0.820					**0.015***
Antrum/Distal	37	30	27	70		32	18	29	35	
Cardia/Proximal, GE junction	9	6	11	19		6	3	1	10	
Fundus/Body	22	13	19	48		7	9	20	31	
**Lauren type**					0.662					**<0.001***
Intestinal	30	19	14	59		29	20	18	23	
Diffuse	2	1	3	5		12	9	34	56	
CMS1	70					45				
CMS2		53					30			
CMS3			58					52		
CMS4				144					80	

*P < 0.05
